# Assessment of PET & ASL metabolism in the hippocampal subfields of MCI and AD using simultaneous PET-MR

**DOI:** 10.1186/2197-7364-2-S1-A73

**Published:** 2015-05-18

**Authors:** Maged Goubran, David Douglas, Steven Chao, Andrew Quon, Pragya Tripathi, Dawn Holley, Minal Vasanawala, Greg Zaharchuk, Michael Zeineh

**Affiliations:** Stanford University, USA

Alzheimer’s disease (AD) has been reported to show decreased metabolic activity in the hippocampus using FDG PET-MR. Histological data suggests that the hippocampal subfields are selectively affected in AD. Given the simultaneous imaging nature of integrated PET-MR scanners and the multimodal capabilities of PET-MR, our purpose here is to assess FDG activity, as well as ASL perfusion in the subfields of MCI and AD patients. 10 consecutive subjects were recruited for this study 3 MCI, 3 AD patients and 4 age-matched controls. The scanning was performed on a simultaneous 3T PET/MR scanner. To delineate the hippocampal subfields, automatic segmentation of hippocampal subfields (ASHS) was employed. Static FDG-PET series were reconstructed for analysis at 45-75 min for all subjects. All imaging sequences were automatically registered to the oblique coronal T2-weighted images (segmentation space). PET standardized uptake values (SUV) in the hippocampal subfields were normalized by the pons. FDG PET metabolism was reduced significantly in AD, as well as MCI patients as compared to controls, with the highest effect demonstrated in the CA3/DG and CA1/2 (p = 0.047, subfields. Patients (MCI and AD combined) had decreased metabolism as compared to controls in CA1/2 and significantly smaller volumes the Subiculum. When assessing CBF across groups, a significant decrease in CBF was found in the Subiculum. Our preliminary results demonstrate that PET-MRI may potentially be a sensitive biomarker and tool for early diagnosis of AD. They also confirm the importance of assessing metabolic and structural changes of neurodegenerative diseases at the subfield level.

